# Thinking beyond otorhinolaryngology in patients presenting with bilateral vocal cord paresis

**DOI:** 10.1093/jscr/rjad140

**Published:** 2023-03-30

**Authors:** Hamza Usman, Faith Protts, Edward Fathers, Edward Chisholm

**Affiliations:** Foundation Doctor at Somerset Foundation Trust, Taunton, UK; Otolaryngology Specialty Registrar at Somerset Foundation Trust, Taunton, UK; Consultant Neurologist at Somerset Foundation Trust, Taunton, UK; Consultant Otolaryngologist, at Somerset Foundation Trust, Taunton, UK

## Abstract

Anti-immunoglobulin-like cell adhesion molecule 5 (IGLON5), a disease first described in 2014 by Sabater *et al*., is characterised by dysphonia, dysphagia, stridor and dysautonomia. We discuss the case of a patient presenting to the emergency department with anti-IGLON5 associated airway compromise following progressive reduced vocal cord movement requiring a surgical tracheostomy. We discuss the outpatient and emergency presentation of this case along with the available literature on anti-IGLON5. We aim to remind ENT practitioners to look beyond the common diagnoses and consider the diagnosis of anti-IGLON5 disease when faced with the symptoms listed above.

## INTRODUCTION

Anti-immunoglobulin-like cell adhesion molecule 5 (IGLON5) is a novel disease first described by Sabater *et al*. [1] and as such awareness of the condition and its complications may be limited, leading to delayed or incorrect diagnosis. In this report, we present the case of a patient diagnosed with anti-IGLON5 and discuss the literature on the disease to help practitioners consider it amongst their differentials when faced with symptoms such as progressive reduced vocal cord movement, dysphagia and obstructive sleep apnoea in patients.

## CASE REPORT

A 74-year-old, Caucasian male, presented to ENT with a 5-month history of dysphonia, mild intermittent dysphagia. Past medical history included of hypertension, obstructive sleep apnoea (OSA) and degenerative arthritis. Examination revealed an asymmetrical laryngeal inlet with reduced right-sided vocal cord movement and supraglottic squeeze. A CT skull base to thorax revealed no structural pathology.

Speech and language team (SLT) review identified ongoing voice change, episodes of dysphagia to dry foods and a single episode of choking on liquids. A diagnosis of muscle tension dysphonia and age-related swallowing difficulties was made and managed with voice and swallowing therapy. Video fluoroscopy swallow assessment demonstrated findings in keeping with the diagnosis.

Though some improvement in symptoms was initially reported, at 7 months, he reported sudden onset of noisy breathing and nocturnal shortness of breath. No audible stridor was noted, flexible laryngoscopy (FL) revealed mildly sluggish movement of vocal cords with breathing noises and voice quality affected by posture. He was allocated patient-initiated follow-up (PIFU) with advice regarding head positioning, ongoing SLT input and safety netting advice.

A month later he was booked for urgent face to face ENT review and review of OSA following worsening of voice and stridor at night following a PIFU telephone consultation.

Before this review occurred, he presented to the emergency department stridulous, with type II respiratory failure and a reduced GCS. Examination revealed persistent bilateral cord adduction. He was transferred to ITU on CPAP, intubated and a surgical tracheostomy was subsequently performed. Repeat CT skull base to thorax and MRI brain looking for structural and neurological causes were unremarkable.

The neurology team were consulted and suggested symptoms may be prodromal of subacute neurological pathology and tested calcium, HIV, treponema serology, Lyme serology, serum protein electrophoresis, acetylcholine receptor antibodies, MuSK antibodies, LRP1 antibodies, which returned normal. The diagnoses highlighted in Table 1 were also considered.

**Table 1:** Differentials considered in our patient that have been reported to cause bilateral vocal cord paresis/paralysis

Differentials	Tests
Tumour	
Lung cancer	CT thorax
Laryngeal cancer	US thyroid/CT skull base to thorax
Brain cancer	CT head
Infection	
*Tuberculosis*	Cultures, sputum acid-fast bacilli smear and nucleic acid amplification
Treponema	Treponema serology
Polio	Refer to local guidelines
Lyme disease	Lyme Serology
Autoimmune	
Myasthenia gravis	Acetylcholine receptor antibodies
Parkinsons	Neurological history, exam and DaTScan
Multi system atrophy	No reliable tests available—refer to diagnostic criteria
Motor neurone disease	MRI brain, electromyopgraphy, nerve conductions studies
Others	
Alcohol-related encephalopathy	CT/MRI head
Cerebrovascular event	CT/MRI head
Organophosphate pesticide toxicity	RBC acetylcholinesterase
Bilateral cricoarytenoid joint fixation	Rheumatoid factor
Laryngeal amyloidosis	Myeloma screen, larynsgoscopy and biopsy

Further neurology review considered a rare cause of stridor, anti-IgLON-5 disease, serum antibodies against IgLON5 returned a positive titre of 1:1000, confirming the diagnosis.

At time of writing, because of no improvement in vocal cord movement, long-term airway support is with a tracheostomy tube. His swallow requires ongoing SLT, and gastrostomy feeding is being considered. Management is currently, supportive with monthly follow up from ENT, Neurology, SLT, respiratory medicine and physiotherapy. Further immunotherapy is being considered.

## DISCUSSION

Anti-IgLON5 disease, first described in 2014, is a neurodegenerative autoimmune disease, which can present over years with sleep disturbances, breathing changes and gait instability [1]. Bulbar symptoms such as stridor, dysphagia and central hypoventilation have been described in 60% of patients [2]. Another review discussed four patients with anti-IgLON5 eliciting evidence of vocal cord paresis on FL [3]. Many reported daytime drowsiness because of sleep disorder and partners complained of abnormal vocalisation and snoring [1, 3]. We can see that multiple bulbar symptoms were present in our patient. Table 2 highlights a variety of other symptoms that Anti-IGLON5 may present with.

**Table 2:** A compilation of various symptoms experienced by patients caused by anti-IGLON5 described in literature [6]

Symptoms of anti-IGLON5	Description
Sleep distrubances	OSA, parasomnia, insomnia, stridor, drowsiness
Bulbar Symptoms	Dysphagia, dysarthria, sialorrhoea, vocal cord paresis, respiratory failure, stidor
Movement disorders	Parkinsonism, chorea
Dysautonomia	Urinary dysfunction, orthostatic hypotension, cardiac dysfunction
Cognitive impairment	May meet criteria of Dementia
Oculomotor abnormalities	Supranuclear gaze palsies

Current hypotheses suggest antibodies against IgLON5, a molecule involved in cell adhesion and neuronal growth regulation, cause irreversible internalisation of the surface IgLON5 protein and an increase in tau protein [2].

The function of these proteins remains unclear [4].

Men and women are equally affected and only 10% of the affected have an autoimmune history [5]. Age at onset ranges from 45 to 83 [5, 6].

Treatment has been attempted with, steroids, plasmapheresis, IVIg, rituximab, azathioprine and mycophenolate [3, 7, 8].

Varying effectiveness of therapy is described in the literature, review of 13 patients, discovered only one showed transient improvement of symptoms [9]. Another, in a case series of 46 patients; 20 responded to immunotherapy; 15 had lasting response to treatment at follow-up [10].

Schröder *et al*. [9] describe a patient in whom plasmapheresis and steroid therapy achieved almost complete resolution of symptoms after presenting with ptosis, vocal cord paresis, facial palsy and dysphagia, and, a subsequent decannulation of tracheostomy.

Combination therapy appears to give best response rates though no consensus exists [7, 10]. Initial studies reported that many patients showed no improvement in symptoms with therapy, though recent suggestion is, treatment specifically within the active phase of disease, may increase efficacy of treatment [10].

Effectiveness of early immunotherapy is highlighted by Grüter *et al*., who describe a patient, demonstrating complete resolution of symptoms following IVIg treatment highlighting the necessity of early disease recognition and treatment; though there continues to be a lack of data, thus, correlations between therapeutic strategy and degree of remaining disease can seldom be drawn [5, 7, 11]. Table 3 highlights the various treatment regimes described in literature.

**Table 3:** Therapies given to patients with anti-IGLON5 as described by recent literature [10]

Monotherapy	Combination Therapy
IV corticosteroids	IVIg + corticosteroid
Rituximab	IVIg + plasmapharesis
IVIg	Corticosteroids + azathioprine
Plasamapharesis	IVIg + mycophenolate mofetil
	IVIg + cyclophosphamide
	IVIg + plasmapheresis + rituximab

In a case series of five patients who presented with symptoms of either OSA, dysphagia or hoarseness, four of these patients went on to require tracheostomies at time of publication [3]. Of these patients, one had their tracheostomy removed because of good treatment response but needed re-insertion because of an acute dyspnoea attack. The other three tracheostomy patients had no further airway complications. Three of the five were also dependent on PEG feed.

Sudden death and aspiration appeared to be the two common causes of death, and death showed no correlation with treatment response [7, 10].

In conclusion, early recognition and treatment could impact disease progression greatly. Given this disease’s varied presentations, we would like to recommend all ENT practitioners consider the diagnosis of anti-IgLON5 disease in the outpatient department when faced with symptoms of dysphonia, dysphagia alongside sleep apnoea and other neurological disturbances in patients.
